# Do Infant Faces Maintain the Attention of Adults With High Avoidant Attachment?

**DOI:** 10.3389/fpsyg.2021.631751

**Published:** 2021-05-07

**Authors:** Nü Long, Wei Yu, Ying Wang, Xiaohan Gong, Wen Zhang, Jia Chen

**Affiliations:** ^1^School of Psychology, Guizhou Normal University, Guiyang, China; ^2^Center for Rural Children and Adolescents Mental Health Education, Guizhou Normal University, Guiyang, China

**Keywords:** infant faces, avoidant attachment state, attentional bias, dot-probe paradigm, facial expression

## Abstract

We investigated whether adults have attentional bias toward infant faces, whether it is moderated by infant facial expression, and the predictive effect of the adult attachment state on it. One hundred unmarried nulliparous college students [50 men and 50 women; aged 17–24 years (*M* = 19.70, SD = 1.35)] were recruited. Each completed a self-report questionnaire—the Chinese version of the State Adult Attachment Measure (SAAM), and a dot-probe task with a stimulus presentation duration of 500 ms, which used 192 black-and-white photographs of 64 people (32 infants and 32 adults; each person displayed three expressions: happy, neutral, and sad) as the experimental stimuli. The results showed that, at the duration of 500 ms, individuals' attentional bias toward infant faces disappeared, regardless of the facial expression. However, when the interaction between avoidant attachment state and face was controlled, the attentional bias was significant again, and the avoidant attachment state negatively predicted individuals' attentional bias toward infant faces. This indicates that at the suprathreshold stage, there are individual differences in the attentional bias toward infant faces, and high avoidant attachment will weaken individuals' attentional bias toward infant faces. This study advances previous studies that focused only on individuals' attention to infant faces occurring at the early processing stage of attention. The results provide direction for interventions; specifically, changing the attachment state of avoidant individuals can affect their attention to infants, which may promote the establishment of parent–child relationships.

## Introduction

Baby schema (or *kindchenschema*) refers to the psychological representation of a specific set of infantile physical characteristics, such as a large head, high forehead, round face, large eyes, small nose and mouth, short and thick extremities, and plump body shape (Lucion et al., 2017). The effect it triggers is a basic social instinct (McClintock and Eibl-Eibesfeldt, 1975). To illustrate, infants must rely on adults' upbringing and care. The baby schema makes adults perceive infants as cute and, thus, generates their affection for infants automatically, which motivates their concern and caretaking behavior (Parsons et al., 2010). This ensures the survival of infants and the reproduction and continuation of the entire population.

Since the concept of baby schema was proposed by Austrian ethologist Konrad Lorenz in 1943 (Lorenz, 1943), many researchers have conducted several studies on its effect, and the results revealed that the face of an infant is the most important representative region of baby schema (Glocker et al., 2009). Therefore, many subsequent studies on baby schema have focused on the babyface schema (Rayson et al., 2017), which provides strong empirical evidence for adults' preference response. This preference response manifests itself in adults' rapid cognitive and behavioral responses to infants' faces (Endendijk et al., 2018), positive emotions (Almanza-Sepdulveda et al., 2018), evaluations (Proverbio, 2017), and a strong willingness to protect and care for infants (Cheng et al., 2015). These studies were of great value in understanding the establishment of human parent–child relationships and promoting good parenting.

Researchers have also examined the mechanism behind this preference and its influencing factors. Among them, some researchers found that attentional bias was an important manifestation of human preference for infants' faces (for a review, see Lucion et al., 2017). Attentional biases refer to a readiness to orient toward and maintain attention on a particular class of stimuli over others (Cisler and Koster, 2010). In 2007, using the dot-probe paradigm, Brosch and his colleagues found that baby schema, being a strong positive stimulus, can trigger humans' attentional bias (Brosch et al., 2007). Since then, attentional bias toward infant faces has received broad attention from researchers. Using different methods such as dot-probe (Li et al., 2019), go/no-go (Dudek and Haley, 2020), and eye-tracking (Jia et al., 2017), among others, with different materials (Rayson et al., 2017) in different populations (Martinez et al., 2020), researchers from diverse countries and cultures all demonstrated that infant faces elicit adults' attentional bias. However, despite this ample research, most current studies only focused on the automatic processing stage, which sets the presentation duration below 240 ms (for a review, see Lucion et al., 2017).

Information processing is generally divided into two stages: automatic and strategic processing (Shiffrin and Schneider, 1977). Automatic processing refers to processing that is effortless, capacity free, unintentional, and outside of conscious control; strategic processing refers to processing that is effortful, capacity limited, intentional, and dependent on conscious control (Shiffrin and Schneider, 1977). In different stages, the pattern of bias was different depending on the duration of exposure to stimuli (O'Toole and Dennis, 2012). Several attentional bias studies (Mogg et al., 1993; Bradley et al., 1999; Koster et al., 2006; Bar-Haim et al., 2007; Torrence et al., 2017) using the dot-probe paradigm have found that although attentional bias toward specific information was found under different stimulus presentation durations (e.g., 17, 50, 100, 240, and 500 ms), when the presentation durations were longer than 300 ms, only difficulty in disengagement was observed (Fox et al., 2002). However, when the durations were presented subliminally, only facilitated attentional orienting was observed (Carlson and Reinke, 2008). Contrastingly, other research (Carlson and Mujica-Parodi, 2015) showed that fearful face threat cues, whether consciously or non-consciously processed, elicit a similar facilitation in orienting and a delayed disengagement of attention. These studies further suggested that attentional bias did not occur in certain stages of information processing. Nonetheless, to our knowledge, whether infant faces can, similar to the threatening stimuli, cause attentional bias in the late attention processing stage remains unelucidated.

In addition to the duration of the stimuli, there was another deficiency in the existing research on attentional bias toward infant faces; that is, most previous research only focused on neutral (Brosch et al., 2007, 2008; Hodsoll et al., 2010) or positive expression (Proverbio et al., 2011) faces; however, infants' expressions are diversified. For infants, whose communication is essentially non-verbal, facial expressions are an important way to communicate information. Therefore, adults need to provide care for infants by accurately processing their emotional expressions and responding appropriately. In some recent studies (Jia et al., 2017), researchers used photos of different adult and infant faces with different expressions and adopted eye-tracking technology to investigate the characteristics of attentional bias toward infants with different expressions. The results showed that attentional bias may change according to different facial expressions. Furthermore, compared with happy and sad expressions, infants' neutral expressions gained the most attentional bias. This may be owing to the uncertainty of neutral expressions, which makes it difficult for adults to recognize them (Cheng et al., 2019).

Therefore, attentional bias studies such as these that consider the facial expressions of infants are necessary. The obvious problem with these studies, however, is that the images of faces they used did not include different expressions (happy, neutral, or sad) from the same individuals, but different expressions from different individuals, which cannot exclude the interference caused by facial structure. However, according to existing studies, babyface schema changes with slight changes in facial structure (Rayson et al., 2017). Therefore, in the above studies, the change of attentional bias cannot be completely explained by different facial expressions because this effect may also be caused by the interference of different facial structures. To investigate whether facial expressions interfere with attentional bias, we must first control for the effect of facial structures, which was also noted in a recent study by Jia et al. (2021). They used a series of images of the same face with multiple expressions to control for facial structure, and the results indicated that attentional bias toward neutral faces was significantly greater than those of happy and sad faces. However, in this study, the researchers also set the stimulus presentation duration to 100 ms, and the authors believed that the maximized baby schema effect of the neutral expression was due to the uncertainty of what the expression suggested. Then, in the suprathreshold stage when the expression is determined, whether the moderate effect of the expression still exists is worth exploring.

As mentioned above, when the duration is short, attentional bias facilitates attentional orienting (Carlson and Reinke, 2008), which is a stage of automatic information processing that is generally less likely to be influenced by individual variables. However, the difficulty in disengagement that occurs when the duration exceeds 300 ms is often considered to involve strategic information processing (Cisler and Koster, 2010), which is controlled by consciousness and may be affected by individual variables. For example, previous studies have confirmed that attentional bias in individuals with high trait anxiety is more susceptible to personality and other factors under the duration of 500 ms than that of non-anxious individuals (Puls and Rothermund, 2018). Therefore, in recent years, the influence of individual variables on attentional bias has also received researchers' attention.

Parents' own attachment style can influence the quality of parental caregiving (Bilge and Sezgin, 2020). A recent study indicated that adult attachment can modulate attentional bias toward infants; women with higher attachment avoidance had less attentional bias for infant faces than women with lower attachment avoidance (Jia et al., 2017). According to the attachment theory, avoidant attachment individuals tend to adopt a deactivation strategy in threatening situations (Schumann and Orehek, 2019). When faced with infant face cues that might activate their attachment system, they adopt ignoring and avoiding strategies to suppress information processing, which reduces psychological tension and anxiety (Gillath et al., 2009a).

However, the empirical research on adult attachment and infant faces attentional bias is very limited. Furthermore, studies on adult attachment in recent years have found that, although attachment style is stable, it may also fluctuate with the establishment of new relationships and experiences (Gillath et al., 2009b). Researchers also found that attachment priming in the lab can lead to temporary changes in individual attachment (Gillath and Karantzas, 2019), and that attachment state is associated with individuals' level of interest in infants (Cheng et al., 2015). Therefore, in addition to stable attachment style, whether this fluctuating attachment state can also predict attentional bias requires further research.

Therefore, in this study, we used a series of images of the same face with multiple expression images of infants and adults to strictly control for the facial structure. Furthermore, we used the dot-probe paradigm to investigate three main questions. First, most previous studies found that attentional bias toward infant faces occurred in the automatic processing stage. Therefore, the question is whether infant faces also play the same role in the late stage of processing. Second, if the attentional bias toward infant face still exists in the late stage of processing, is it moderated by facial expression? Furthermore, can the conclusions proposed by previous studies—that infants' neutral facial expressions gained the most attentional bias—be verified again? Third, can attachment states predict attentional bias toward infant faces under different facial expression conditions? In addition, according to existing studies (Cardenas et al., 2013), there may be sex differences in attentional bias toward infant faces; however, other researchers (Brosch et al., 2007; Jia et al., 2021) found that sex differences were not significant. Therefore, in this study, we also included the gender variable. Specifically, we proposed the following three hypotheses: (1) at the durations of 500 ms, adults will have attentional bias toward infant faces; (2) facial expressions will moderate attentional bias toward infants, and neutral infant facial expressions will garner the most attentional bias; and (3) adult attachment state and gender will predict individuals' attentional bias toward infant faces.

## Methods

### Power Analysis

We used a 2 (faces: infant or adult) × 3 (expressions: happy, neutral, or sad) × 2 (men or women) mixed design. A power analysis was performed to determine the sample size using the “Power Analysis for General Anova” tool (PANGEA; Westfall, 2016; Judd et al., 2017). Specifically, a custom design was specified with the following four factors: participants (random and crossed), faces (fixed and crossed), expressions (fixed and crossed), and sex (fixed and nested). The purpose was to detect interaction among faces, expressions, and sex based on three specified parameters (the others were set to default values). The parameters were a typical effect size (*d* = 0.258) in a meta-analysis of attentional bias for positive emotional stimuli (Pool et al., 2016), the number of observations per given experimental condition for each participant in the current design (128 replicates), and the estimated sample size. This analysis indicated that a sample size of 66 participants would provide 95% power. Considering the insufficient number of applicants and loss of participants, in order to ensure 95% power, we planned to recruit 100 participants and prepare 192 stimuli.

### Participants

One hundred participants (50 men and 50 women; all right-handed and with normal or corrected-to-normal vision) from Guizhou Normal University were recruited through a psychological course. All were unmarried and nulliparous, aged 17–24 years (*M* = 19.70, SD = 1.35), and reported no diagnosed history of communicative, cognitive, or attentional disorders.

All participants provided written informed consent to participate. Participation was anonymous, and each participant received 20 RMB as compensation. They were told that they could withdraw from the study at any time without reason. The study met the ethical requirements of the American Psychological Association and the Declaration of Helsinki. It was also approved by the ethics committee of the authors' university (no. 2014179).

### Measure

The Chinese version (Ma et al., 2012) of the State Adult Attachment Measure (SAAM; Gillath et al., 2009b) was used to capture individual differences in temporary fluctuations of attachment. It contained three reliable subscales measuring state levels of attachment-related anxiety, avoidance, and security. Participants responded to 21 items using a seven-point Likert scale ranging from 1 (*disagree strongly*) to 7 (*agree strongly*), with 4 (*neutral/mixed*) as the midpoint. Contrary to the original measure, which had seven items for each subscale, the Chinese version had five items for anxiety and nine for security. The avoidance subscale was identical for both versions. The score of each SAAM subscale was generated by summing the item scores of each subscale. A higher score indicated higher attachment tendency of the corresponding dimension. Furthermore, this Chinese version of the SAAM had excellent psychometric properties (Ma et al., 2012). In this study, the internal consistencies for anxiety, avoidance, and security subscales were 0.682, 0.735, and 0.718, respectively.

### Stimuli

The experimental stimuli consisted of 192 black-and-white photographs of 64 people (32 infants: sex-neutral, aged 0.25–1 year; 32 adults: 16 men and 16 women, aged 18–27 years). Each person displayed three expressions: happy, neutral, and sad. All photographs were taken from a multi-expression image database for infants and adults (Jia et al., 2019). Subsequently, the hair, ears, and background were cropped from each image, leaving a series of facial outlines. Any non-face area of the image region (260 × 300 pixels) was filled with a black background (RGB: 0, 0, 0).

All these facial stimuli were matched for size, luminance, and contrast. There were 96 adult–infant face-pairs. There were no significant differences in emotional intensity (emotional intensity and the recognition of each facial stimuli were from the image database) between the infant stimuli and adult stimuli [happy: *t*_(62)_ = 1.193, *p* = 0.237; neutral: *t*_(62)_ = 0.844, *p* = 0.402; sad: *t*_(62)_ = 1.447, *p* = 0.153], and the recognition rates were high for all expressions (*M* = 0.914, SD = 0.099).

## Procedure

The experiment was conducted in a quiet laboratory room in the department. To ensure the test quality, we performed our procedure with six participants at a time, based on participant availability. Participants were instructed to complete the Chinese version of the SAAM (Ma et al., 2012) and the computer task, which was programmed by E-Prime 2.0. To counterbalance the effect of questionnaire and task sequencing, half of the participants completed the questionnaire first, and the other half completed the task first. The whole procedure lasted approximately 40 min.

In the task, participants sat in a comfortable chair facing the computer screen (47.6 cm × 26.8 cm) at a distance of 100 cm. The task instructions were presented in writing on the screen and explained orally. Then, participants were asked to complete 36 practice trials, which required an accuracy of 90% or more (if <90%, they practiced again). The formal experiment consisted of four identical blocks including 108 experimental trials with a 1-min rest among the blocks. Following a previous procedure that used the dot-probe task with threatening stimuli (Lipp and Derakshan, 2005), in each trial, a face pair of an infant and an adult face was presented. One face was shown to the left (*x*: 240, *y*: center) and the other was shown to the right (*x*: 1,040, *y*: center) of the fixation.

**Figure 1:**
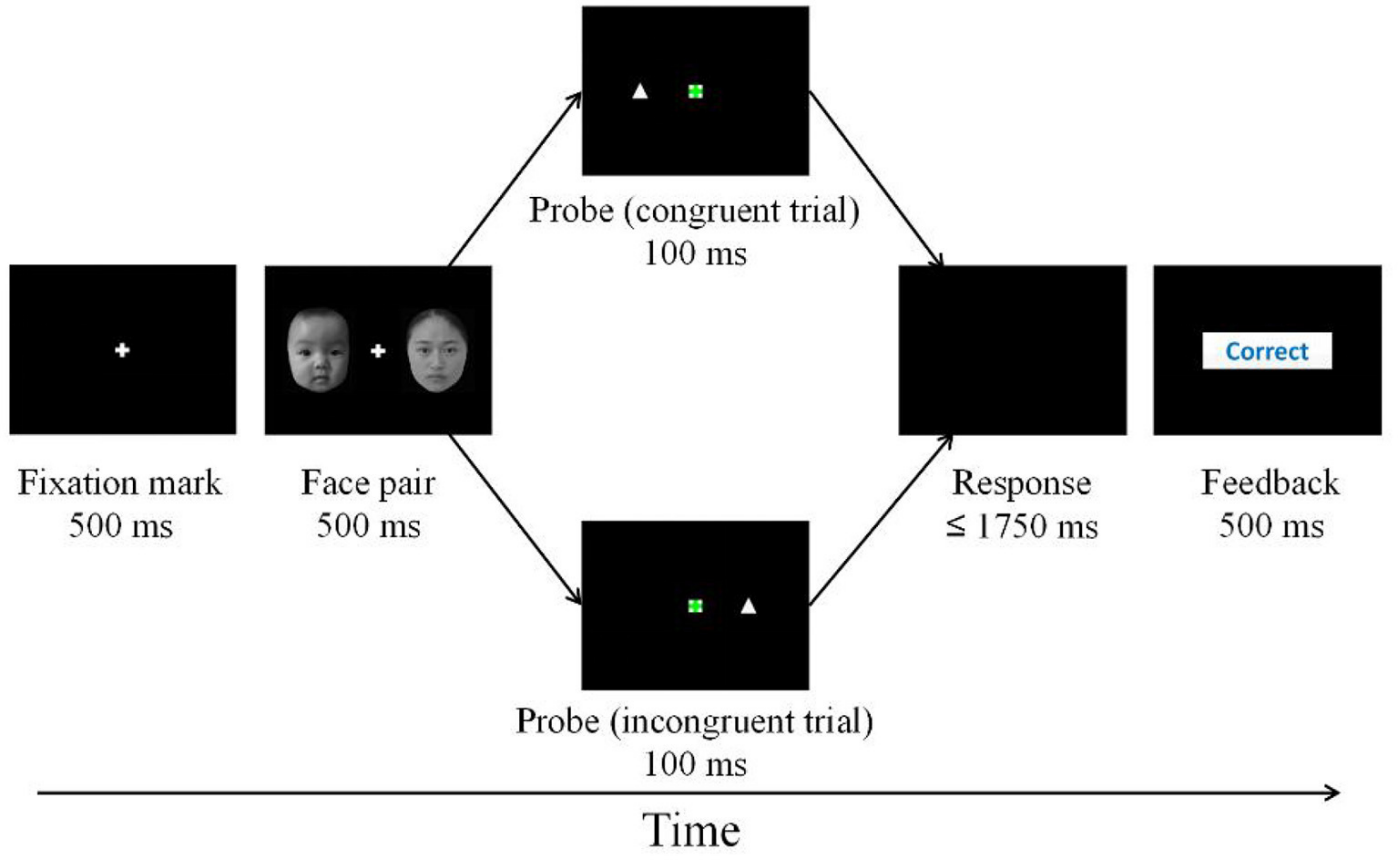
Example order of individual trial sequence for the dot-probe task.

A trial started with the presentation of a white fixation cross for 500 ms in the center of the screen. This was followed by presenting a face pair horizontally aligned, also for 500 ms. Then, the fixation cross turned different colors (from white to green or red). The dot-probe (a white equilateral triangle) appeared for 100 ms, replacing one of the faces (in a congruent trial, the dot replaced the infant face; in an incongruent trial, the dot replaced the adult face). If the cross turned red, participants were required to press the “space bar”; if it turned green, they were required to identify the direction of the equilateral triangle and press the corresponding keys (“H” for upturned or “B” for downturned) as quickly (within 1,750 ms) and as accurately as possible. Following each response, accuracy feedback was presented where either blue “correct” or red “incorrect” was displayed in the center of the screen for 500 ms. Then, the next trial began (see Figure 1).

Participants completed 432 experimental trials. To prevent participants from developing response strategies, 48 (12 trials per block) of the trials were followed by a red cross, and these trials were not entered in the final analysis. The other 384 trials (96 trials per block) were followed by a green cross. Each block of 96 trials consisted of four repetitions of the 24 possible combinations with the following counterbalanced variables: expression pair type (sad, neutral, or happy), the position of the infant face (left or right of the fixation cross), the target type (upturned or downturned), and the congruence (dot replaced the infant face or dot replaced the adult face). Trials in each block were presented in a random order for each participant. After completing the dot-probe task and the questionnaire, participants were informed of the study purpose.

## Statistical Analyses

### Examination of Accuracy and Preparation of Data

All participants' accuracies were over 90%, and the mean accuracy (i.e., correctly identifying the color of the cross and the direction of the probe) was 97.82%. Only the response times of correct responses were used in the final analysis (Salemink et al., 2007). In addition, reaction times (RTs) below 200 ms and those that were 2.5 standard deviations above each participants' individual median (Thigpen et al., 2018) were eliminated.

### Calculation of Attentional Bias

To examine the relationship between adult attachment state and preference for infants, an index of attentional bias (*D*) was calculated by subtracting the mean RT for infant faces from the mean RT for adult faces: *D* = RTa – RTi. Here, RTa represents the mean RT of incongruent trials: dots replacing adults; RTi represents the mean RT of congruent trials: dots replacing infants. A positive *D* score indicated a faster response to dots appearing after infant faces, as compared with adult faces (Salemink et al., 2007). In sum, positive scores indicated more infant bias, and negative scores indicated more adult bias.

### Analyses

All statistical analyses were performed using SPSS 26.0 (Statistical Software, Inc., Chicago, IL, USA). The linear mixed-effects models (LMMs) were used instead of the repeated-measures analysis of variance (ANOVA) to reveal the differences among participants. In the preprocessing of variables, dummy coding was performed for sex (0 = male, 1 = female), and the scores of three attachment states (i.e., secure, avoidance, and anxiety) were converted into *Z*-scores.

First, to verify the existence of the attentional bias toward infants, and whether this bias was influenced by facial expressions and participants' sex, an LMM of RT as a dependent variable was developed with three fixed variables (sex, faces, and expressions) and one random variable (participants).

Second, if the fixed effects of faces or expressions were significant, the aforementioned attentional bias (*D*) index was calculated to represent the degree of attentional bias toward infant faces with collapsing three expressions or each expression.

Third, to explore the attentional bias toward infant faces with collapsing three expressions or each expression and whether it was influenced by various adult attachment states, an LMM of RT as a dependent variable was developed with five fixed variables (face, sex of participants, and three adult attachment states) or six fixed variables (face, expressions, sex of participants, and three adult attachment states).

To estimate the observed effect sizes, partial eta squared ($\eta_{\text{p}}^{2}$), Cohen's *d*_s_, and Cohen's *d*_z_ were calculated for *F*-tests of the LMMs' fixed effects, independent *t*-tests of the faces and expressions and participants' sex differences, and dependent *t*-tests of multiple comparisons with Bonferroni correction (Lakens, 2013). Significance was set at 5% for all analyses.

### Modeling Approach

According to the practice guidance for LMMs (Brysbaert, 2007; Meteyard and Davies, 2020), the modeling approach involved specification, selection, and comparison.

First, for the dependent variable of RT, a model specification in SPSS was designed with the subject variables (participants), repeated variables (faces and expressions/face or face and expressions), repeated covariance type (alternative), random covariance type (variance components), and estimation (all parameters as defaults). The idea behind this model specification was to accurately capture participants' RT in response to infant and adult faces with different expressions by controlling the random variance between participants.

Second, given the theoretical interest, the premise of model selection was to keep seven fixed effects (sex, faces, expressions, and all their interactions)/nine fixed effects (sex, face, the sex-by-face interaction, three attachment states, and each attachment state-by-face interactions) or 11 fixed effects (sex, face, expression, the sex-by-face interaction, the expression-by-face interaction, three attachment states, and each attachment state-by-face interactions) unchanged. The random-effect structure was selected with a “minimal to maximal converges” modeling process from the minimal random-effect structure (an intercept for participants) to a larger one (an intercept for participants, slopes for expressions or for the faces-by-expressions interaction or for faces and expressions or for expressions and the faces-by-expressions interaction or for face and the faces-by-expressions interaction) to the maximal one (an intercept for participants, slopes for faces, expressions, and the faces-by-expressions interaction)/the minimal random-effect structure (with an intercept for participants) to a larger one (an intercept for participants, slopes for three attachment states or for each attachment state-by-face interactions) to the maximal one (an intercept for participants, slopes for three attachment states, and each attachment state-by-face interactions). For each random-effect structure, all alternative repeated covariance types were tested.

Third, the model comparison was based on the restricted maximum likelihood (REML) estimation and the convergence of the alternative models. As an exploratory study, the Akaike information criterion (AIC) was regarded as the primary criterion for model comparison (Aho et al., 2014). A smaller value of AIC indicated a better model fitting for the data when the alternative models had the same random-effect structure. For the nested models with different random-effect structures, a chi-square likelihood ratio test (LRT) was used to test the null hypothesis stating that the smaller model provided as good a fit for the data as the larger model (Matuschek et al., 2017). If the null hypothesis was rejected, the larger model provided a significant improvement over the smaller model.

The full syntax, outputs, and results are included in the Supplementary Material (10.6084/m9.figshare.13728274), in which the final best-fitting model is marked in bold and was reported.

## Results

Table 1 shows the mean RTs in the dot-probe task. It indicates that the mean RTs to images of infants were faster than the mean RTs to images of adults in all expression pair types, regardless of sex.

**Table 1:** Descriptive statistics for RT data from the dot-probe task (*M* ± SD).

**RT to probes (ms)**		**Trial type**	
**Stimuli**		**Congruent**	**Incongruent**
Happy pair	Men	448.12 ± 55.45	452.20 ± 57.88
	Women	484.13 ± 53.53	487.97 ± 49.99
	Total	466.12 ± 57.16	470.09 ± 56.73
Neutral pair	Men	448.81 ± 55.02	449.35 ± 53.56
	Women	484.14 ± 50.34	486.67 ± 53.35
	Total	466.48 ± 55.38	468.01 ± 56.39
Sad pair	Men	448.17 ± 55.38	449.57 ± 55.29
	Women	482.12 ± 48.88	483.47 ± 49.73
	Total	465.14 ± 54.70	466.52 ± 55.02
Total		465.92 ± 55.57	468.21 ± 55.88

*Congruent represents dots replacing infants; incongruent represents dots replacing adults. RT, reaction time; ms, milliseconds*.

The “type III tests of fixed effects” table (Table 2) shows that there was a significant main effect of face, *F*_(1,247.00)_ = 10.39, *p* = 0.001, η_*p*_^2^ = 0.04. However, the difference [i.e., attentional bias index (*D*)] between the adult (*M* = 468.21, SE = 5.27) and infant (*M* = 465.92, SE = 5.27) faces was quite small (only 2.29 ms); thus, the effect may be very low. Moreover, the “estimates of fixed effects” table (Table 3) shows that when the other terms entered in the model were controlled for, the main effect of faces was no longer significant, *t*_(488.12)_ = 0.73, *p* = 0.465. In addition, no significance was found in the main effect of expression and all interactions. This suggested that the attentional bias toward infant faces had disappeared in the whole group at this processing stage.

**Table 2:** Type III tests of fixed effects results for sex, face, expression, and their interactions.

**Fixed effects**	***df*1**	***df*2**	***F***	***p***	**η^**2**^**
Intercept	1.00	98.00	7,893.43	0.000	0.99
Sex	1.00	98.00	11.32	0.001	0.10
Face	1.00	247.00	10.39	0.001	0.04
Expression	2.00	423.05	2.95	0.053	0.01
Sex × face	1.00	247.00	0.16	0.690	0.00
Sex × expression	2.00	423.05	0.88	0.415	0.00
Face × expression	2.00	439.35	1.26	0.284	0.01
Sex × face × expression	2.00	439.35	0.19	0.828	0.00

**Table 3:** Estimates of fixed effects in the final best-fitting model results for sex, face, expression, and their interactions.

**Fixed effects**	**β**	**SE**	***df***	***t***	***p***	**95% CI**
Intercept	482.12	7.53	103.29	64.00	0.000	467.18 to 497.06
Male	−33.96	10.65	103.29	−3.19	0.002	−55.08 to −12.83
Adult	1.35	1.85	488.12	0.73	0.465	−2.28 to 4.99
Happy	2.01	1.84	452.36	1.09	0.275	−1.60 to 5.62
Neutral	2.02	1.95	346.47	1.03	0.302	−1.82 to 5.86
Adult × male	0.05	2.62	488.12	0.02	0.985	−5.09 to 5.19
Happy × male	−2.05	2.60	452.36	−0.79	0.430	−7.16 to 3.05
Neutral × male	−1.37	2.76	346.47	−0.50	0.620	−6.81 to 4.06
Adult × happy	2.49	2.52	326.40	0.99	0.325	−2.47 to 7.45
Adult × neutral	1.17	2.77	312.17	0.42	0.672	−4.28 to 6.63
Adult × happy × male	0.19	3.57	326.40	0.05	0.957	−6.82 to 7.21
Adult × neutral × male	−2.04	3.92	312.17	−0.52	0.603	−9.75 to 5.68

However, considering that at the duration of 500 ms, this is already a top-down processing (Cisler and Koster, 2010), which is controlled by consciousness, individual differences, especially the aforementioned attachment state, may have influenced the attentional bias toward infant faces. Furthermore, since there was no interaction or main effect of expression, to simplify the model, we calculated the total mean RTs to adult and infant faces while collapsing three expressions to further investigate whether attachment state has a moderating effect on attentional bias toward infant faces. Table 4 shows the mean attentional bias index (*D*) and three attachment states. Moreover, the results of the LMM (see Tables 5, 6) showed that after controlling for attachment and its interaction with faces, there were significant main effects of face, *F*_(1,95.00)_ = 10.29, *p* = 0.002, η_*p*_^2^ = 0.10, β = 2.66, *t*_(95.00)_ = 2.63, *p* = 0.010, and sex, *F*_(1,95.00)_ = 11.90, *p* = 0.001, η_*p*_^2^ = 0.11, β = −36.31, *t*_(95.87)_ = 3.41, *p* = 0.001. The interaction effect of faces and avoidant state was also significant, *F*_(1,95.00)_ = 5.35, *p* = 0.023, η_*p*_^2^ = 0.05.

**Table 4:** Descriptive statistics for attentional bias index (*D*) and attachment state.

		***M* ± SD**	**Range**
Attentional bias index (*D*)	Men	2.01 ± 6.66	−16.36 to 15.62
	Women	2.57 ± 7.77	−12.71 to 18.72
	Total	2.29 ± 7.20	−16.36 to 18.72
Security	Men	46.74 ± 7.96	19.00 to 59.00
	Women	47.82 ± 7.36	31.00 to 63.00
	Total	47.28 ± 7.65	19.00 to 63.00
Avoidance	Men	21.36 ± 7.75	7.00 to 41.00
	Women	22.20 ± 7.95	7.00 to 40.00
	Total	21.78 ± 7.83	7.00 to 41.00
Anxiety	Men	21.40 ± 4.79	11.00 to 35.00
	Women	22.06 ± 5.92	9.00 to 34.00
	Total	21.73 ± 5.37	9.00 to 35.00

*Index of attentional bias (D) is calculated by subtracting the mean RT for congruent trials from the mean RT for incongruent trials. Positive scores indicate more infant bias, and negative scores indicate more adult bias. RT, reaction time*.

**Table 5:** Type III tests of fixed effects results for sex, face, attachment states, and their interactions.

**Fixed effects**	***df*1**	***df*2**	***F***	***p***	**η^**2**^**
Intercept	1.00	95.00	7,811.04	0.000	0.99
Sex	1.00	95.00	11.90	0.001	0.11
Face	1.00	95.00	10.29	0.002	0.10
Sex × face	1.00	95.00	0.27	0.603	0.00
*Z*-security	1.00	95.00	0.69	0.409	0.01
*Z*-avoidance	1.00	95.00	1.87	0.175	0.02
*Z*-anxiety	1.00	95.00	0.15	0.696	0.00
Face × *Z*-security	1.00	95.00	0.45	0.504	0.00
Face × *Z*-avoidance	1.00	95.00	5.35	0.023	0.05
Face × *Z*-anxiety	1.00	95.00	0.93	0.337	0.01

**Table 6:** Estimates of fixed effects in the final best-fitting model results for sex, face, attachment states, and their interactions.

**Fixed effects**	**β**	**SE**	***df***	***t***	***p***	**95% CI**
Intercept	484.07	7.51	95.87	64.43	0.000	469.16 to 498.98
Male	−36.31	10.66	95.87	−3.41	0.001	−57.46 to −15.15
Adult	2.66	1.01	95.00	2.63	0.010	0.65 to 4.68
Adult × male	−0.75	1.44	95.00	−0.52	0.603	−3.60 to 2.10
*Z*-security	−4.81	6.15	95.87	−0.78	0.436	−17.02 to 7.40
*Z*-avoidance	−7.14	5.91	95.87	−1.21	0.230	−18.87 to 4.59
*Z*-anxiety	1.92	5.90	95.87	0.33	0.746	−9.79 to 13.63
Adult × *Z*-security	−0.56	0.83	95.00	−0.67	0.504	−2.20 to 1.09
Adult × *Z*-avoidance	−1.84	0.80	95.00	−2.31	0.023	−3.42 to −0.26
Adult × *Z*-anxiety	0.77	0.80	95.00	0.96	0.337	−0.81 to 2.35

**Figure 2:**
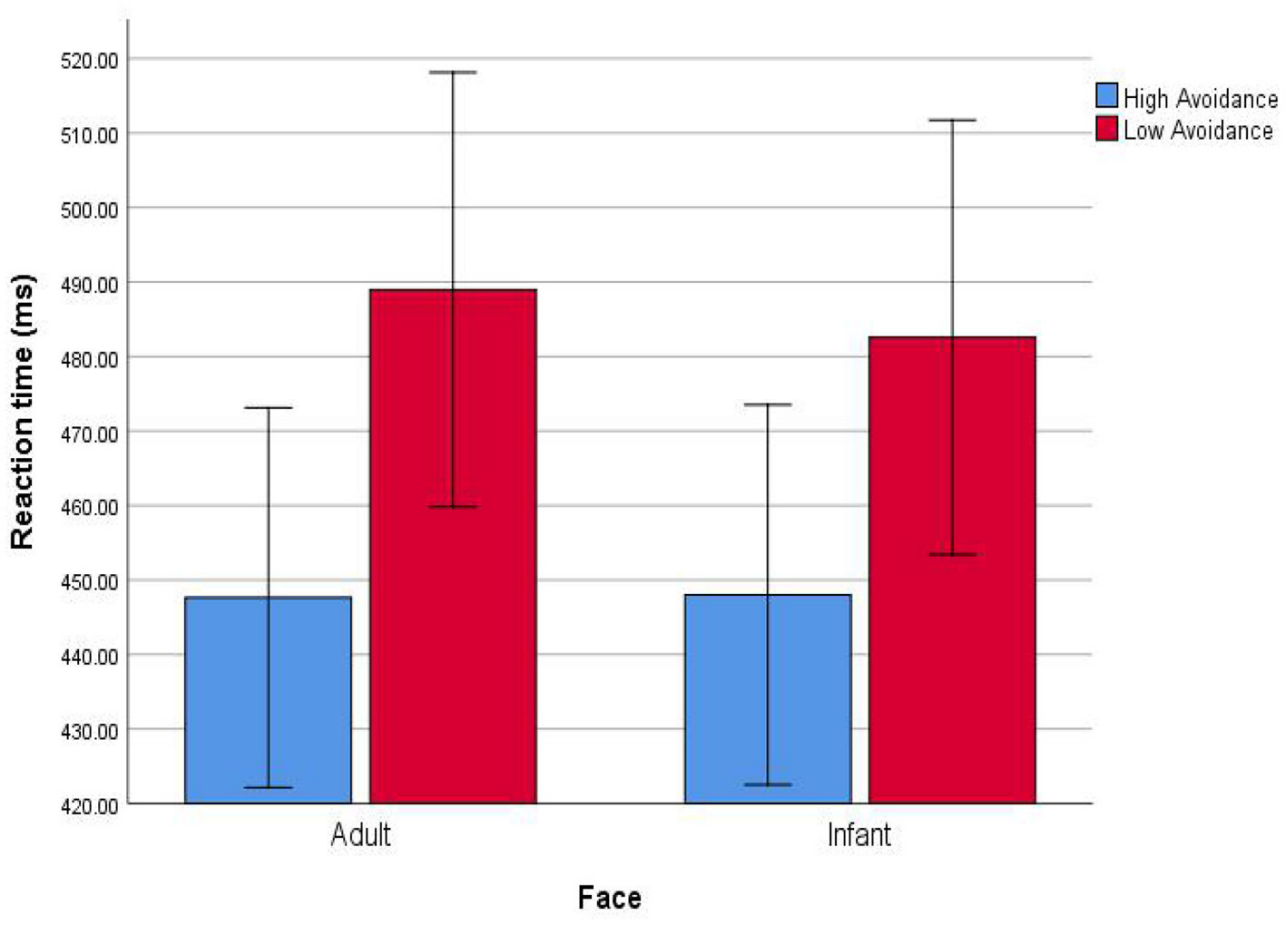
Individuals' avoidant attachment state moderates the effect of attentional bias toward infant faces. Error bars are 95% confidence intervals.

To facilitate the interpretation of this interaction effect, we divided the individuals into high and low groups according to the scores of avoidant attachment state, and then conducted a simple slope test (Dearing and Hamilton, 2006). The results are depicted in Figure 2, which shows that there was no difference (i.e., attentional bias) in RT of high avoidant attachment individuals between infant and adult faces. They were quick to respond to all faces. However, compared with adult faces, low avoidant attachment individuals have stronger attentional bias toward infant faces, such that when the infant face appeared, their reactions were faster. The results of the estimate of fixed effects indicated that avoidant attachment negatively moderated attentional bias toward infant faces, β = –1.84, *t*_(95.00)_ = −2.31, *p* = 0.023.

A *post-hoc* test for the RTs of faces revealed that after controlling for attachment and its interaction with faces, RTs to images of adults (*M* = 468.21, SE = 5.30) were significantly longer than RTs to images of infants (*M* = 465.92, SE = 5.30), *t*_(95.00)_ = 3.21, *p* = 0.002, Cohen's *d*_z_ = 0.32. A *post-hoc* test for the RTs of sex indicated that men (*M* = 448.72, SE = 7.50) responded faster than women (*M* = 485.40, SE = 7.50), *t*_(98.00)_ = 3.45, *p* = 0.001, Cohen's *d*_s_ = 0.69.

## Discussion

Inconsistent with the results of previous studies (Brosch et al., 2007; Martinez et al., 2020; Jia et al., 2021) using dot-probe paradigms to explore attentional bias toward infant faces, the results of this study indicated that individuals' attentional bias toward infant faces disappeared. However, most previous researchers set the stimulus presentation durations within 240 ms (for a review, see Lucion et al., 2017), which mainly inspected the attentional bias occurring at the automatic stages of processing. In this study, the duration of stimulus presentation was extended to 500 ms. Koster et al. (2006) found that the duration of stimulus presentation of 500 ms, that is, the attentional bias to suprathreshold presentation, was an indicator of both automatic and strategic processing. At this stage, the processing gradually turned from effortless, unintentional, and non-conscious to effortful, intentional, and dependent on conscious control, which is affected by personality variables (Puls and Rothermund, 2018). In addition, considering that the difference between the three expressions was non-significant, we collapsed three expressions to further investigate whether attachment had a moderating effect on individuals' attentional bias toward infant faces at this stage.

The results showed that after controlling for the interaction between attachment and face, the main effect of face was significant again, and the avoidant attachment state negatively moderated the attentional bias of individuals, i.e., individuals with high avoidant attachment state had a weaker attentional bias toward infant faces. This means that at 500 ms, there were individual differences in the attentional bias toward infant faces, which resulted in the disappearance of attentional bias in general.

Therefore, the attentional bias toward infant faces at 100 ms confirmed by previous studies (Brosch et al., 2007; Jia et al., 2021) may be due to the absence of interference from attachment at the automatic processing stage. Therefore, no matter whether the level of the avoidant state was high or low, the attentional bias would not be affected. At 500 ms, however, when attachment took effect, the attentional bias disappeared in the high avoidant individuals, while the low avoidant individuals still had the attentional bias toward infant faces. Moreover, this exactly meant that at the early stage of automatic processing, possibly due to the baby schema effect, infant faces first attract our attention without our effort, and then, at the later stage of strategic processing, infant faces maintain our attention depending on our conscious control.

This was congruent with Swain's (2011) parental brain model. The human parental brain is a concept of a discrete set of interacting brain circuits that serves as a substrate for the human transition to parenting, integrating baby stimuli as well as internal information, and supporting key thoughts and behaviors for us to identify and react to baby stimuli (Swain, 2011). According to the working model of Swain's human parental circuits, key parenting sensory signals, such as a baby crying, visuals, and touching and smelling a baby, are first organized into sensory cortices, which appraise the input and interact with subcortical memory and motivation structures. Subsequently, the corticolimbic modules are activated. These consist of three parts. The first part is reflexive caring impulses, which require little or no cortical input. Obviously, individuals' attention to an infant is an automatic and unconscious process in this part. The second part is the cognitive circuits, which are under conscious control, including those that regulate mirroring, empathy, planning, and further cognitive flexibility that may allow individuals to accurately predict infants' needs. Therefore, individuals' attention to infants in this part may have gradually risen to the level of consciousness. Finally, other alarm/emotion–preoccupation anxiety systems might be activated to increase arousal and regulate parental worries and habitual responses in coordination with memory systems. In this part, the individual may be more inclined to pay attention to the infant since the emotional module was activated; thus, they generate the behavioral output required for parenting.

Hence, the human parental brain ensures that humans' recognition and response to infants' stimuli have a certain specificity, which makes it easier for the infant stimuli to acquire an individual's attention system. As soon as an individual attends to the infant stimuli, their attention is maintained. That is, attraction to infant stimuli may increase attentional maintenance, resulting in a longer response time in disengaging attention away from infant stimuli. This is consistent with previous studies (Posner et al., 1987; Theeuwes, 2010; Theeuwes and Belopolsky, 2012; Torrence et al., 2017) stating that attentional bias can be driven by two mechanisms: (a) early attentional mechanisms, such as initial orienting toward the stimulus, that appear rapidly (i.e., before 120 ms after the stimulus onset) and are conceived as independent of the observer's intentions, or (b) later attentional mechanisms, such as difficulty in disengaging one's attention from the stimulus and reallocating it toward another stimulus that appear more slowly (i.e., 200–250 ms after the stimulus onset), which are more likely to be influenced by voluntary processes. Therefore, possibly due to the initial orienting, infant faces can make us more easily or quickly attracted to them; subsequently, due to the difficulty in disengaging that occurs later, they can also make us maintain our attention on them, while making it difficult in reallocating it toward another stimulus. However, the specific components of attentional bias in different stages need to be confirmed in future studies.

It is worth noting that, in our experiment, adult faces aged 18–27 were used as the stimuli, while our participants were also young adults aged 17–24. The infant faces may be more novel to them relative to faces of young adults, which may lead to our participants' attentional bias toward infant faces. However, all face materials (whether adult faces or infant faces) we used in our experiment were unfamiliar to the participants. While young adults may see more faces of their peers, infant faces are also very common in our daily lives, such as on TV, in advertisements, in pictures, in everyday life, and so on. According to the infant schema effect, an infant face is considered to have a unique facial structure (Glocker et al., 2009). Moreover, since the concept of baby schema was proposed, many relevant empirical studies in this field have confirmed that, in addition to young adults, infant faces can also cause attentional bias of individuals in other age groups (Pearson et al., 2011; Thompson-Booth et al., 2013, 2018). Therefore, it is evident that attentional bias toward infant faces, to a large extent, was not caused by the novelty of infant faces to young adults. However, in future studies, adult face images with a large age span should be used to further verify this claim and solve the possible impact of age characteristics on the experimental results.

However, inconsistent with previous studies (Jia et al., 2021) proposing the maximization effect of a neutral babyface schema, our study did not find a moderating effect of facial expression on attentional bias toward the infant. From the facial expression-level analysis, our study showed that, under the stimulus presentation duration of 500 ms, the difference between the three expressions was non-significant, that is, the attentional biases of the adults toward the three expressions of infant faces were consistent. The reason for these inconsistent results may be that facial expressions moderate attention differently at different processing stages. Furthermore, previous studies (Jia et al., 2021) set the stimuli presentation duration at 100 ms; thus, they examined adults' attentional bias toward infant faces that occurred at the early stage. At this stage, possibly due to the neutral expression being more ambiguous than the happy and sad expressions, individuals displayed delayed recognition of infants' neutral expressions (Jia et al., 2021). This suggests that the maximization effect of a neutral babyface schema may only exist at the early processing stage. Contrastingly, at the late stage of attention maintenance, the facial expression had already been identified; thus, the delayed effect was eliminated, and individuals showed consistent attention maintenance to the various expressions of infant faces. This finding, however, requires further empirical studies to confirm.

Existing studies on threat stimuli indicated that conscious strategic processing is affected by several personality variables (Puls and Rothermund, 2018). Among them, adult attachment has an important influence on emotional and cognitive mechanisms—sustaining individual interpersonal relationships, attention, and so on (Sagliano et al., 2014). In this study, we found that the infant face, as a positive stimulus, can also cause some individuals' attentional bias toward it, but this attentional bias was moderated by attachment state. Here, the attachment state of avoidance was a significant predictor of attentional bias toward infant faces. For individuals with low avoidant attachment, the attentional bias persisted, while for those with high avoidant attachment, the attentional bias began to disappear.

This result may be related to the “internal working modes” of individuals. According to Griffin and Bartholomew's (1994) self-model/other-model system, which was based on Bowlby's (1977) concept of working models of the self and other, attachment patterns result from different combinations of positivity or negativity of self- and other-models. Many empirical studies have confirmed the strong relationship between negative other-models and avoidance (Otani et al., 2018). Avoidant individuals tend to be more self-reliant and believe that others are unreliable and untrustworthy; therefore, they have a strong sense of threat toward interpersonal communication. Thus, they reject and escape from all dependent needs and physical and emotional intimacy, and they rarely empathize with others (Fraley, 2019).

Under the influence of the negative other-models, when processing attachment-related information, they always tend to adopt the deactivating strategy to escape the painful experience caused by the activation of the attachment system. These deactivating strategies have an important impact on attentional processes (Otani et al., 2018). For instance, avoidant individuals in romantic relationships can inhibit attention to negative and positive attachment-related material (Schumann and Orehek, 2019). Furthermore, some studies found that avoidant individuals had a certain attentional avoidance of emotional faces (Yulisha et al., 2016). Thus, as illustrated in this study, for individuals with high avoidant attachment, their bias toward infant faces may be suppressed or weakened to a certain extent owing to the abovementioned inner working model. Therefore, avoidant attachment negatively predicted individuals' attentional bias toward infant faces.

This coincides with actual parenting behavior. Studies of parenting found that, compared with their counterparts, avoidant individuals tend to avoid opportunities to be close to children, care less for children, and have weaker emotional connections with children (Parsons et al., 2010). These factors may have a negative effect on an individual's parenting ability. A previous study that adopted eye-tracking technology also verified that individuals with higher attachment avoidance may lack attentional preference for infant faces (Jia et al., 2017). Jia et al. explored the influence of attachment styles on individual attentional bias; however, attachment styles are stable and not easily altered (Raby and Dozier, 2019). Recently, other researchers found that attachment state can fluctuate with the establishment of new relationships or the experience of new life events (Mikulincer and Shaver, 2020).

Some studies even used priming stimuli to activate individuals' secure or insecure attachment state in an attachment-priming paradigm (Gillath and Karantzas, 2019). Attachment state is a better indicator of our current attachment level than attachment style. Moreover, the SAAM used in this study can effectively capture the temporary fluctuations of attachment style with a change in situation and relationship. According to our results, in addition to avoidant attachment style, avoidant attachment state can also effectively predict individuals' attentional bias toward infant faces. Thus, the results suggested that, if attachment state can be changed by an intervention, it may affect the maintenance of attention to infants by reducing the avoidant state of avoidant individuals. This is of great significance in establishing good parent–child relationships.

In recent years, with the expansion of research on attachment changes, it was found that insecure attachment had plasticity (Gillath and Karantzas, 2019), which can inform clinical interventions. In the future, to prevent unhealthy parent–child relationships, family interventions can be used to reduce parents' avoidant attachment state so that they will be more willing to pay attention to their children and make more eye contact with them. This may also affect parent–child interactions and improve parental quality. Although previous research (Cardenas et al., 2013) has found that women have a stronger attentional bias for infant faces, this was not found in our study. Moreover, our study only showed that men responded faster than women. This may be due to men showing a greater degree of cerebral lateralization toward the right hemisphere for both processing faces (Proverbio et al., 2006) and positive facial expressions (Bourne, 2005). Furthermore, none of the interactions involving sex reached statistical significance in our study; thus, further research needs to examine the potential gender differences in the perception of infant faces as found in previous studies.

This study showed that at the suprathreshold stage, individuals' attentional bias toward infant faces began to disappear, but this disappearance is moderated by individuals' attachment state. Although our results help elucidate the relationship between adult attachment and infant face attentional bias, there were some limitations. First, this study only used a scale to measure individuals' attachment state and concluded that avoidant attachment can predict individuals' attentional bias toward infant faces. However, since we did not activate the attachment state operationally, we cannot infer causal conclusions. In future research, it is necessary to further investigate whether there is a covariant relationship between individuals' attachment state and attentional bias toward infant faces by priming individuals' attachment state. Second, the novelty effect of infant faces may be caused by our experiment stimuli, which may have influenced the results. In future studies, adult face images with a large age span should be used for further verification and solving the impact of age characteristics on experimental results. In addition, this study did not investigate the interaction between adults and infants in real situations, and we only investigated the attentional bias of nulliparous, unmarried individuals. Therefore, whether the results are applicable to parents and their own children in real situations requires exploration.

## Data Availability Statement

The datasets presented in this study can be found in online repositories. The names of the repository/repositories and accession number(s) can be found in the article/Supplementary Material.

## Ethics Statement

The studies involving human participants were reviewed and approved by the Human Subjects Review Committee of Guizhou Normal University. Written informed consent to participate in this study was provided by the participants' legal guardian/next of kin. Written informed consent was obtained from the individual(s), and minor(s)' legal guardian/next of kin, for the publication of any potentially identifiable images or data included in this article.

## Author Contributions

NL and JC contributed to the conception and design of the study. WY, YW, and XG organized the database. NL and WZ performed the statistical analysis. NL wrote the first draft of the manuscript. All authors contributed to manuscript revision, read, and approved the submitted version.

## Conflict of Interest

The authors declare that the research was conducted in the absence of any commercial or financial relationships that could be construed as a potential conflict of interest.
